# Association between removable prosthesis-wearing and pneumonia: a systematic review and meta-analysis

**DOI:** 10.1186/s12903-024-04814-5

**Published:** 2024-09-11

**Authors:** Tong Wah Lim, Kar Yan Li, Michael Francis Burrow, Colman McGrath

**Affiliations:** 1 Division of Restorative Dental Sciences, Faculty of Dentistry, The University of Hong Kong, Pok Fu Lam, Hong Kong, Hong Kong SAR; 2 Dental Public Health, Faculty of Dentistry, The University of Hong Kong, Sai Ying Pun, Hong Kong, Hong Kong SAR; 3 Division of Applied Oral Sciences and Community Dental Care, Faculty of Dentistry, The University of Hong Kong, Hong Kong, Hong Kong SAR

**Keywords:** Pneumonia, Removable denture, Respiratory tract infections, Systematic review

## Abstract

**Background:**

A high burden of respiratory pathogens colonizing removable prosthesis surfaces suggests the potential of association between removable prosthesis-wearing and respiratory infections. Therefore, this systematic review and meta-analysis aimed to evaluate the evidence from clinical studies concerning the association between removable prosthesis-wearing and respiratory infections.

**Methods:**

Clinical studies that reported respiratory infections associated with adult patients wearing removable prostheses in any centers (hospitals and nursing homes) or communities were included. Literature was searched across five electronic databases (MEDLINE, Cochrane Library, EMBASE, Web of Science, and Scopus) to 28 May 2024. An additional search was performed for unpublished trials and references cited in related studies. The Newcastle-Ottawa Scale was employed for the quality assessment. The certainty assessment was established using GRADE. The results were pooled using a frequentist random-effects meta-analysis and the odds ratios generated.

**Results:**

A total of 1143 articles were identified. Thirteen articles had full-text articles screening and an additional two articles were added through reference linkage. Ultimately, six non-randomized clinical studies reporting various types of pneumonia contributed to this review. Overall odds of having pneumonia among prosthesis wearers were 1.43 (95% CI: 0.76 to 2.69) and 1.27 (95% CI: 1.11 to 1.46) using the random- and fixed-effects models, respectively. The heterogeneity in the meta-analysis was substantial. In subgroup analysis according to the study design, the heterogeneity within prospective studies was much reduced, I^2^ = 0% (*p* = 0.355). The certainty of the evidence evaluated using the GRADE approach was low to very low evidence for prosthesis wearers developing pneumonia based on studies.

**Conclusions:**

There was no conclusive evidence from the non-randomized clinical studies supporting whether prosthesis-wearing is a risk factor for pneumonia based on outcomes from this review.

**Supplementary Information:**

The online version contains supplementary material available at 10.1186/s12903-024-04814-5.

## Background

The association between opportunistic pathogens in the oral cavity and respiratory diseases has been gaining popularity in the dental and medical fields [1–4]. One of the most reported respiratory diseases, pneumonia, is the leading cause of morbidity and mortality among older people globally. The incidence of pneumonia varies among countries, community-dwelling, and institutionalized people, and increases rapidly with age [3]. It is well known that most pneumonia is partly caused by bacteria through micro-aspiration, which has been strongly correlated with the oral bacterial species. For aspiration pneumonia, it can be also caused by macro-aspiration of a mixture of oral bacteria including the commensal flora and oropharyngeal secretions containing pathogenic microorganisms [4, 5]. Notably, aspiration pneumonia should be considered as part of community-acquired pneumonia and hospital-acquired pneumonia [4]. Some studies [6–10] revealed the possible relationship between removable denture-wearing and respiratory infections, particularly denture-wearing was reported to have a 7-fold higher risk associated with community-acquired pneumonia compared with the non-denture group. Prosthesis-wearing at night and infrequent prosthesis cleaning were found significantly associated with pneumonia [6, 8]. In addition, denture stomatitis was also found as a key factor (increased odds ratio 5.71) associated with bacterial pneumonia [10]. Therefore, faced with a rapidly aging population, the impact of wearing removable prostheses on respiratory diseases cannot be disregarded [4, 11].

The high prevalence of prosthesis wearers among elders also possesses a high risk of aspirating respiratory pathogens from the prosthesis biofilm into their respiratory system due to the proximity of the prosthesis to their respiratory tract [12, 13]. Appropriate prosthesis hygiene is of paramount importance to care and reduce the risk of pneumonia and other opportunistic infections [14–16]. In hospital settings, poor prosthesis hygiene of removable prosthesis wearers was found to be significantly associated with postoperative pneumonia [8]. In addition, the fungi and bacteria on prosthesis surfaces are reported to trigger secondary coinfections and aggravate existing lung infections, resulting in longer hospitalization times and a higher risk of death [17]. Furthermore, many studies have reported a high burden of respiratory pathogens present on removable prostheses [12, 15, 16, 18]. Bacteria and fungi from prosthesis biofilm were also reported as potential sources of infection in patients diagnosed with chronic obstructive pulmonary diseases [19]. Therefore, it is no surprise that these respiratory pathogens may demonstrate their pathogenic potential in at-risk patients, particularly older adults with underlying comorbidities, compromised mucociliary functions, and a decreased host immune system [2]. In contrast, Takeuchi et al. [20] reported that partially edentulous patients who wear prostheses experience increased salivary secretion which improves the self-cleansing ability of the prosthesis and oral cavity. Thus, results in a reduction of respiratory pathogens’ colonization intraorally and is thus beneficial for patients with a risk of aspiration pneumonia, particularly individuals with dysphagia [21]. Therefore, the relationship between wearing removable prostheses and respiratory diseases remains uncertain.

Though two systematic reviews [22, 23] exist evaluating the contribution of poor oral health to pneumonia, there is only one study [24] included in one of the reviews assessing prosthesis-wearing as a risk factor. Additionally, a recent systematic review [15] reported a high burden of respiratory pathogens colonizing removable prosthesis surfaces suggesting the association between removable prosthesis-wearing and respiratory infections should be further investigated. There is an urgent need to evaluate an exact estimate of the risk and need to increase in the understanding of the role of removable prosthesis-wearing in the development of respiratory diseases by a systematic appraisal of the evidence. Hence, the present systematic review aimed to evaluate the evidence from clinical studies concerning the association between prosthesis-wearing and respiratory diseases.

## Materials and methods

### Registration and protocol

This review was registered under the PROSPERO, International prospective register of systematic reviews (ID: CRD42022361983), and reported in compliance with the guidelines set forth by the Preferred Reporting Items for Systematic Reviews and Meta-Analyses (PRISMA) (Additional file 1) [25].

### Eligibility criteria

The inclusion criteria were established according to population, exposure, comparison, outcome, and study (PECOS) schema. However, no patient or public involvement in this review. MeSH terms and free keywords in the search strategy were also defined based on the same approach. Studies were considered eligible according to the following inclusion and exclusion criteria:

P: This review focussed on adult patients aged 18 years old and above (mean) with no restriction on their health condition, from any centers (hospitals and nursing homes) or communities.

E: Removable prosthesis-wearing regardless of the type of prosthesis (partial or complete design; acrylic resin or cobalt-chromium framework) or hygiene status was considered as exposure.

C: No removable prosthesis-wearing was the control.

O: The outcome was defined as respiratory diseases including pneumonia, chronic obstructive pulmonary disease, cystic fibrosis, coronavirus disease, and asthma diagnosed by medical care providers or charted in medical records. For pneumonia, community- and hospital-acquired pneumonia were used to describe the origin of the infectious agents derived from the community and hospital setting (acquired at least 48 to 72 h after admission), respectively.

S = This review was limited to clinical studies including clinical controlled trials, cross-sectional, case-control, and cohort studies, excluding literature and systematic reviews, protocols, in vitro studies, and case reports. Studies evaluating pneumonia after any oral care intervention were also excluded as this review focused on a risk factor (prosthesis-wearing) rather than intervention.

### Information sources and search strategy

Literature was searched across five electronic databases including MEDLINE (PubMed), EMBASE, Scopus, Web of Science, and the Cochrane Central Register of Controlled Trials (CENTRAL). The search strategy employed for the aforementioned databases is presented in Additional file 2. This search strategy was verified by a dental and medical librarian of the Faculty of Dentistry, The University of Hong Kong. A manual search of the references cited in relevant reviews and full-text articles was conducted to identify any additional related studies that might have been missed. Additional searching was also performed for unpublished trials using ClinicalTrials.gov and the International Clinical Trials Registry Platform. The literature search stage was completed on May 28, 2024. There were no restrictions on the year of publication, and only studies in the English language were included in this review.

### Selection process

Endnote X9 software (Thompson Reuters, Philadelphia, PA, USA) was used to manage the imported articles identified through electronic database searches and duplicates were removed. The initial identification of articles, title and abstract screening, and assessment of eligibility were carried out independently by two reviewers (TWL and MMA). Full-text versions of all potentially relevant studies were obtained for further assessment. Any disagreements concerning the eligibility of included studies were addressed through discussion and a third reviewer (CM) was consulted to resolve discrepancies if consensus could not be reached.

### Data collection process and data items

Two reviewers (TWL and MMA) independently gathered and organized data from the included studies using a standardized collection spreadsheet (Microsoft Excel 365, Microsoft Corporation, Redmond, WA, USA). Afterward, the completed spreadsheets were compared, and any disagreements were resolved through consensus. The extracted information included a narrative synthesis of the findings from included studies, structured around all the characteristics of the studies including the name of the authors, year of publication, country, methods (study design, center, inclusion period), participants (number of subjects and dropout, age of subjects, type of prosthesis), outcome measures (type of respiratory disease and diagnostic criteria), results related to prosthesis-wearing associated with respiratory disease (odds ratios and their corresponding 95% CIs). Corresponding authors were contacted in cases of missing data.

### Study risk of bias assessment

The quality of the included studies was evaluated independently by two reviewers (TWL and MMA) using the Newcastle-Ottawa Scale (NOS) [26]. This scale contains eight items for three categories: (1) selection of the study groups; (2) comparability between groups; and (3) evaluation of the exposure of interest, with a total maximum score of nine points. Any disagreements between the reviewers regarding the assessment were resolved by consensus, with the involvement of a third reviewer (CM) if required.

### Effect measures and synthesis methods

The results were pooled using a frequentist random effects meta-analysis, the Procedure Metaprop_one with cimethod (exact) option, Stata 16.0 (StataCorp. 2019. Stata Statistical Software: Release 16. College Station, TX: StataCorp LLC.). This method computed the study-specific confidence intervals using the exact method while the confidence intervals of the pooled estimate were estimated by the Wald method. The odds ratio was calculated from the number of events that happened and did not happen in both the exposed (removable prosthesis-wearing) and unexposed (non-removable prosthesis-wearing) groups if the direct values were not provided. The combined estimate was calculated as the weighted average of the estimates in the individual studies. Heterogeneity was assessed using the I^2^ statistic and Chi-square, I^2^ > 50% and *p* < 0.1 were considered indicative of substantial heterogeneity while I^2^ > 90% implied considerable heterogeneity. Subgroup analyses were performed for types of pneumonia (community- and hospital-acquired) and study designs (prospective, retrospective, and case-controlled), to explore the heterogeneity. However, subgroup analyses for countries and centers of the study were not conducted per protocol due to inadequate data available. Sensitivity analysis was conducted by comparing fixed- and random-effects meta-analyses.

### Reporting bias assessment

The Begg test [27] was planned to be conducted if there were 10 or more studies included in this systematic review, in order to assess the publication bias.

### Certainty assessment

In the present review, Grading of Recommendations, Assessment, Development and Evaluation (GRADE) was employed to establish confidence, evaluate the quality of the included evidence, and summarise the findings using GRADEpro GDT [28]. The certainty assessment considered the following domains: (i) study design, (ii) risk of bias, (iii) inconsistency, (iv) indirectness, (v) imprecision, and (vi) other considerations. Evidence certainty was categorised into four grades, ranging from very low to high, reflecting our confidence in the effect estimation and its adequacy to support a particular recommendation. If disagreements arose between the reviewers, they were resolved through consensus, and if required, a third reviewer (CM) was involved.

## Results

### Study selection

**Fig. 1:**
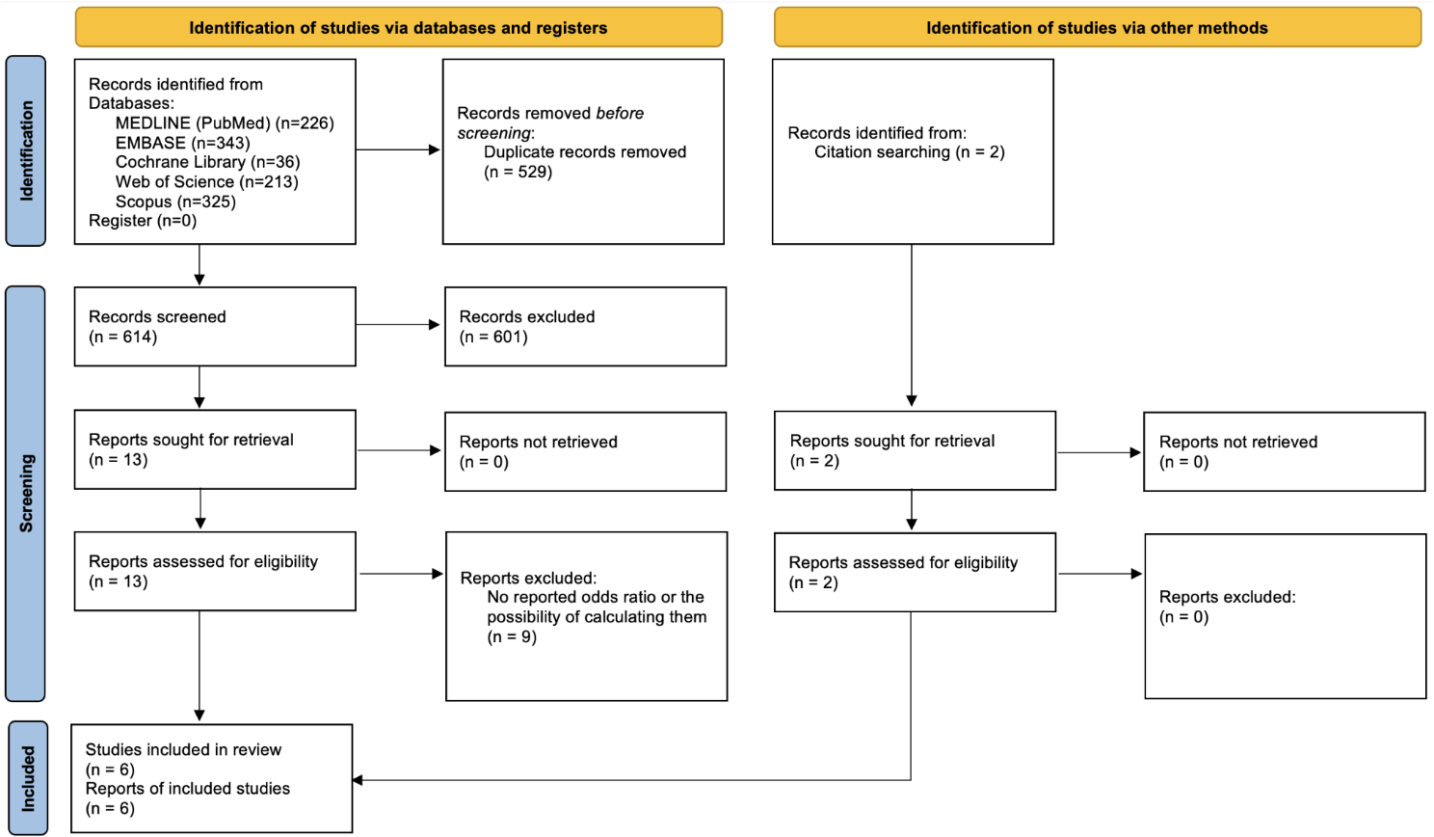
Flow diagram for selection of articles

A total of 614 articles were identified after duplicates were removed through the electronic database search. Thirteen studies were considered for full-text review after the title and abstract screening stage. A hand search yielded two additional articles. After applying inclusion and exclusion criteria, a total of six non-randomized studies reporting pneumonia were included for qualitative analysis. Excluded studies after full-text reading are presented with reasons in Additional file 3. A meta-analysis assessing the association between prosthesis-wearing and pneumonia was performed for them. The Cohen’s kappa coefficient (κ) for the full-text articles screening was 0.84, indicating considerable agreement between the two reviewers (Fig. 1).

### Study characteristics

Among all six included studies [9, 24, 29–32], five cohorts (2 prospective and 3 retrospective) and one case-control study. The included studies were published between 2007 and 2021, three from Europe, two from Japan, and one from the United States. Four studies recruited subjects aged 65 and above, the remaining two ranged from 54 to 60. The total number of participants involved in the analysis was 5879, ranging from 90 to 2498. The odds ratio for the association of prosthesis-wearing with pneumonia ranged from 0.45 (95% CI: 0.24 to 0.84) to 5.42 (95% CI: 2.91 to 10.11), and the mean follow-up time ranged from 3 months to 8 years. There was one study that reported removable prosthesis wearing significantly reduced the risk of aspiration pneumonia. Notably, the prosthesis hygiene and prosthesis-wearing habits of participants in this study were revealed as satisfactory [30]. The types of pneumonia that were reported included community-acquired (3 studies), hospital-acquired (1 study), and a mixture of both (2 studies). Among them, two were diagnosed with aspiration pneumonia [30, 31]. The pneumonia was diagnosed by physicians using various criteria and investigations. Detailed characteristics of the included studies are presented in Table 1.

**Table 1:** Characteristics of the included studies

Authors and year of publication	Country	Methods			Participants			Respiratory tract infections		Outcome related to removable prosthesis wearing.
		Study design	Centre	Inclusion period	Subject (Dropout)	Age (years)	Type of removable prosthesis	Type	Diagnostic criteria	
[24]	United Kingdom	Prospective Follow-up: 17 months	Glasgow Royal Infirmary	2004–2005	Patients with stroke: 412 (8) Prosthesis: 211	67	Partial or complete: NA Acrylic or cast base: NA Mandible or maxilla: NA	Community & Hospital-acquired pneumonia	Diagnosis was performed by assessing the medical records using Mann criteria pneumonia.	The presence of removable prostheses was not significantly associated with pneumonia after stroke.
[23]	Spain	Case-control Follow-up over 1 year	Public primary care centres and regional hospitals	1999–2000	Patients with community-acquired pneumonia: 1336 (52.9% men; 47.1% women) Control: 1326 (52.6% men; 47.4% women)	Patients with community-acquired pneumonia: Males (58.6); Females (54.6) Control: Males (58.9); Females (54.6)	Partial or complete: NA Acrylic or cast base: NA Mandible or maxilla: NA	Community-acquired pneumonia	Diagnosis was performed based on criteria for clinical suspicion of acute lower respiratory tract infection, atypical community-acquired pneumonia, and pneumonia for elderly patients. All cases were verified with chest radiographs.	Dental prosthesis is positively associated with community-acquired pneumonia but is not a strong association. OR, 1.22; 95% CI, (1.04 to 1.42)
[31]	Japan	Retrospective Follow-up: 1 year	Patients admitted to the Departments of Neurology or Stroke Medicine in the past.	1996–2005	Patients with stroke: 143 − 103 men − 40 women	72 (median)	Partial or complete: NA Acrylic or cast base: NA Mandible or maxilla: NA	Community & Hospital-acquired aspiration pneumonia	Diagnosis was performed by the physician based on fever ≥ ºC and chest radiograph.	Presence of prostheses were positively associated with the onset of aspiration pneumonia: (OR, 2.843; 95% CI, 1.011 to 8.048)
[29]	United Kingdom	Prospective Follow-up: 3 months	Orthopaedic wards, General hospital	2009–2010	93 patients with lower limb fracture (3 dropout) (66 prosthesis wearers)	65–101	Partial or complete: NA Acrylic or cast base: NA Mandible or maxilla: NA	Hospital-acquired pneumonia	Diagnosis was performed by the responsible clinician using American Thoracic Society and British Society for Antimicrobial Chemotherapy guidelines, with chest radiographs.	Prosthesis wearing (OR, 1.71; 95% CI, 0.34 to 8.68)
[32]	United States	Retrospective Follow-up: 8 years	Patients at the University of Rochester Medical Center	2010–2018	Prosthesis: 1189 Without prosthesis: 1175	> 57	Partial (37.24%) or complete (45.44%) or combination (17.32%) Acrylic (62.43%) or cast base (37.57%) Mandible (12.05%) or maxilla (24.10%) or both (63.85%)	Community-acquired pneumonia	Diagnosis was performed by the study patients’ medical care providers and charted in the medical records.	Prosthesis wearing significantly increased the risk of pneumonia among the older adults. (HR, 7.68; 95% CI, 3.91 to 15.08) after adjusting for covariates.
[30]	Japan	Retrospective Follow-up: 2 years	Community-based integrated care unit	2018–2020	412 − 138 men − 274 women	86.9	Partial or complete: both Acrylic or cast base: NA Mandible or maxilla: both	Community-acquired aspiration pneumonia	Diagnosis was performed by physician based on clinical symptoms, chest radiograph or computed tomography images, and blood test findings within 48 h of admission.	Prosthesis wearing significantly reduced the risk of aspiration pneumonia in the hospital. (OR, 0.360; 95% CI 0.172 to 0.754) Notably, prosthesis hygiene and prosthesis-wearing habits for participants in this study were reported as satisfactory.

NA, not available; OR, odds ratio; HR, hazard ratio; CI, confidence interval

### Risk of bias in studies

For risk of bias assessment, a score of 7 stars on the NOS was recorded for 4 cohort studies [24, 29–31] and 1 case-control study [32] as shown in Tables 2 and 3. There was one cohort study [9] that was rated 8 stars on the NOS, which all were considered as good quality (Table 2). The main reasons for reducing the quality of evidence were lacking ‘comparability of cohorts on the basis of the design or analysis’ for cohort studies and ‘outcome assessment’ for the case-control study, respectively.

**Table 2:** Quality assessment of cohort studies, Newcastle-Ottawa quality assessment scale

	Coding item	[24]	[31]	[29]	[9]	[30]
	Selection					
1.	Representativeness of the exposed cohort	*	*	*	*	*
2.	Selection of the non-exposed cohort				*	
3.	Ascertainment of exposure	*	*	*	*	*
4.	Demonstration that outcome of interest was not present at start of study	*	*	*	*	*
	Comparability					
1.	Comparability of cohorts on the basis of the design or analysis	*	*	*	*	*
	Outcome					
1.	Assessment of outcome	*	*	*	*	*
2.	Was follow-up long enough for outcomes to occur	*	*	*	*	*
3.	Adequacy of follow up of cohorts	*	*	*	*	*
Global scale		*******	*******	*******	********	*******

*, star; A maximum of one ‘star’ for each item within the ‘Selection’ and ‘Outcome’ categories; maximum of two ‘stars’ for ‘Comparability’

**Table 3:** Quality assessment of case-control study, Newcastle-Ottawa quality assessment scale

	Coding item	[23]
	Selection	
1.	Is the case definition adequate?	*
2.	Representativeness of the cases	*
3.	Selection of Controls	*
4.	Definition of Controls	*
	Comparability	
1.	Comparability of cases and controls on the basis of the design or analysis	*
	Exposure	
1.	Ascertainment of exposure	*
2.	Same method of ascertainment for cases and controls	*
3.	Non-Response rate	
Global scale		*******

*, star; A study can be awarded a maximum of one ‘star’ for each numbered item within the Selection and Exposure categories. A maximum of two ‘stars’ can be given for Comparability

### Results of individual studies and syntheses

**Fig. 2:**
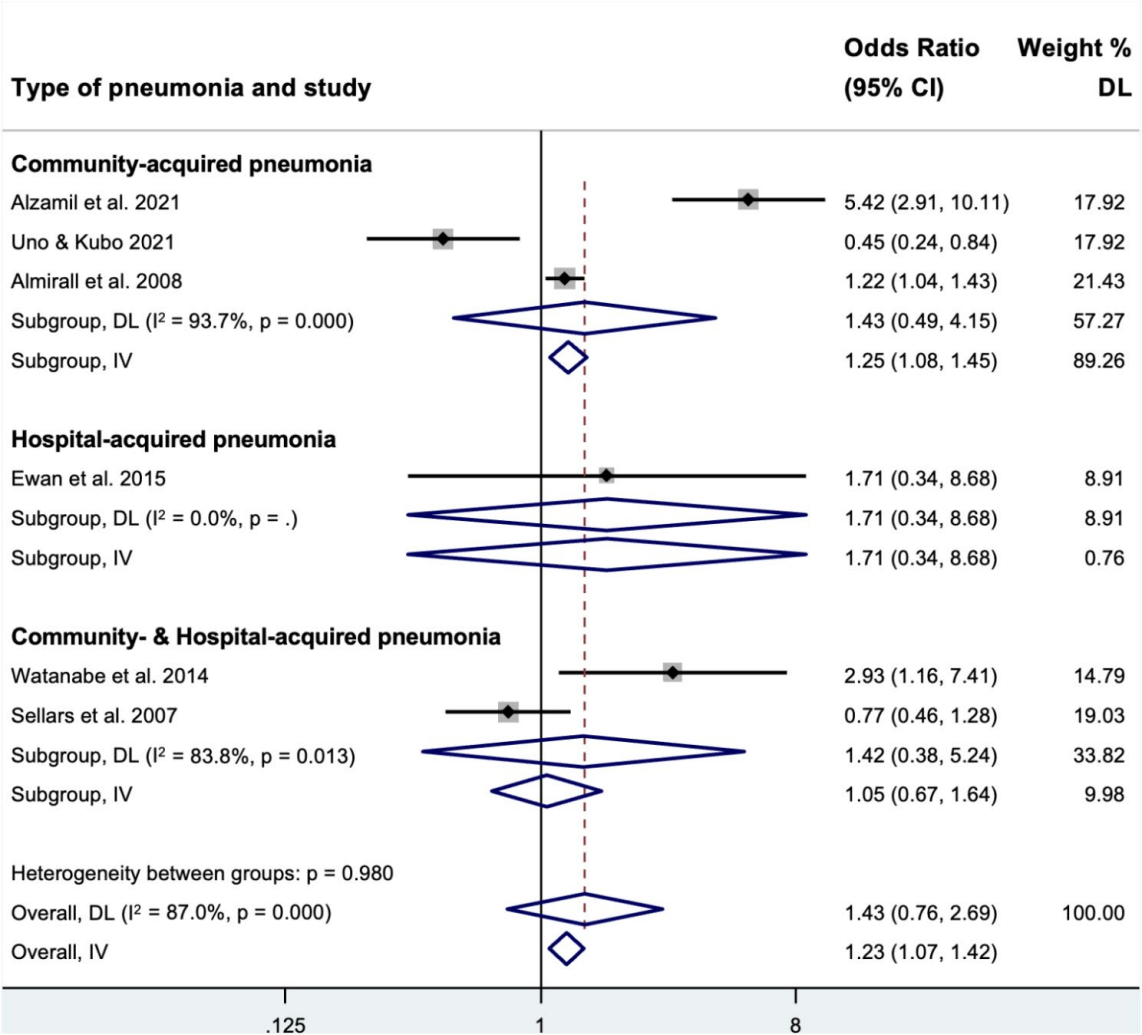
Forest plot of the estimated odds ratio for different types of pneumonia using random-effects (DL, DerSimonian and Laird) and fixed-effects (IV, Inverse Variance) models

Six studies were included for quantitative analysis to evaluate the association between prosthesis-wearing and pneumonia with the mean follow-up time ranging from 3 months to 8 years. Overall odds ratios for the entire sample were 1.43 (95% CI: 0.76 to 2.69) and 1.23 (95% CI: 1.07 to 1.42) using the random- and fixed-effects model, respectively (Fig. 2). For the types of pneumonia subgroup analysis, the odds ratio was estimated as 1.43 (95% CI: 0.49 to 4.15) for community-acquired pneumonia using the random-effects model, and 1.25 (95% CI: 1.08 to 1.45) using the fixed-effects model. However, the overall heterogeneity in this review and heterogeneity in subgroup ‘community-acquired pneumonia’ were substantial (I^2^ = 87.0%; *P* < 0.001) and considerable (I^2^ = 93.7%, *P* < 0.001), respectively.

**Fig. 3:**
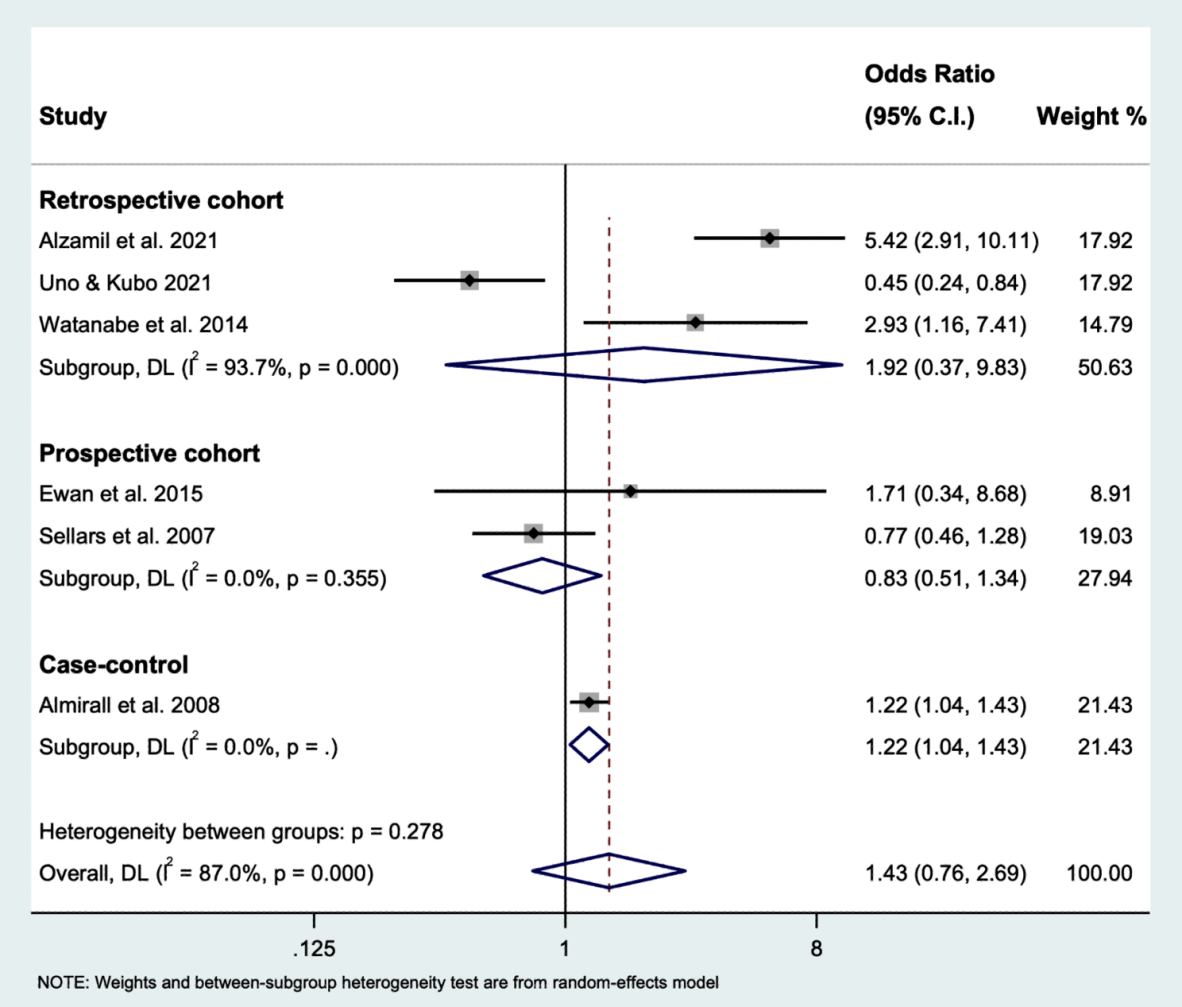
Forest plot of the estimated odds ratio of pneumonia based on study design using random-effects model

Subgroup analysis according to study design (prospective, retrospective, and case-control) showed that the heterogeneity for the subgroup ‘retrospective’ was considerable, I^2^ = 93.7% (*P* < 0.001). While the heterogeneity for the subgroup ‘prospective’ was lowered to ‘might not be important’, I^2^ = 0% (*P* = 0.355) (Fig. 3). In the retrospective cohort group, the estimated odds ratio was 1.92 (95% CI: 0.37 to 9.83). In the prospective cohort group, the estimated odds ratio was 0.83 (95% CI: 0.51 to 1.34). The only case-control study reported that prosthesis-wearing was significantly associated with pneumonia, with an odds ratio of 1.22 (95% CI: 1.04 to 1.43).

### Sensitivity analysis and reporting bias

Sensitivity analysis was performed using fixed- and random-effects meta-analyses. There was a significant association between pneumonia and prosthesis-wearing using fixed-effects meta-analysis, with an odds ratio of 1.27 (95% CI: 1.11 to 1.46). However, the overall odds ratio was 1.43 (95% CI: 0.76 to 2.69) using random-effects meta-analysis. Notably, no statistical analysis was performed to detect publication bias using Begg’s methods, as only six studies were included in the meta-analysis.

### Certainty of evidence

Among the included studies, the certainty of evidence generated from the GRADE approach presented low (case-control) to very low (cohort) levels of evidence due to findings of inconsistency and imprecision as shown in Table 4.

**Table 4:** GRADE assessment

Certainty assessment							№ of patients		Effect		Certainty	Importance
№ of studies	Study design	Risk of bias	Inconsistency	Indirectness	Imprecision	Other considerations	Denture-wearing	Non-denture-wearing	Relative (95% CI)	Absolute (95% CI)		
**Pneumonia**												
5	Observational studies (cohort)	Not serious	Serious^a^	Not serious	Very serious^b^	None	134/1706 (7.9%)	99/1675 (5.9%)	**RR 1.47** (0.61 to 3.54)	**28 more per 1,000** (from 23 fewer to 150 more)	⨁◯◯◯ Very low	CRITICAL
**Pneumonia**												
1	Observational studies (case-controlled)	Not serious	Not serious	Not serious	Not serious	None	1079 cases 1419 controls		**OR 1.22** (1.04 to 1.43)	-	⨁⨁◯◯ Low	CRITICAL
							-	50.0%		**50 more per 1,000** (from 10 more to 88 more)		

CI: confidence interval; OR: odds ratio; RR: risk ratio

**Explanations**

a. The overall heterogeneity (I square) was substantial (*p* < 0.001) and minimal or no overlap of confidence intervals

b. Wide confidence interval

## Discussion

The present review aimed to assess the association between prosthesis-wearing and respiratory diseases, including pneumonia, chronic obstructive pulmonary disease, cystic fibrosis, coronavirus disease, and asthma. However, only pneumonia was included due to the odds ratios for prosthesis-wearing related to other respiratory tract infections could not be generated. Although two published systematic reviews [22, 23] have identified poor oral health as a risk factor for pneumonia, the association between removable prosthesis-wearing and pneumonia remains unknown. Recently, various studies showed a high prevalence of respiratory pathogens residing on removable prostheses [12, 15, 16, 18]. However, published clinical studies investigating the association of respiratory infections with prosthesis-wearing were scarce as shown in the current systematic review.

The quality assessment for cohort and case-control studies was performed using NOS. In general, all included studies were considered good quality. However, the heterogeneity (I^2^ statistic) among the studies was high, 87%, which was considered substantial heterogeneity. The subgroup analysis according to the study design showed the heterogeneity within prospective studies was reduced to 0%, while heterogeneity for retrospective cohort studies was still considerably high, this might be due to study designs, dropout rates, differences in study subjects, and differences in interventions [33]. This also suggested one of the heterogeneity sources was because of the great variations of the study designs among the included studies in this review. Upon sensitivity analysis, the estimated odds ratios showed a significant and insignificant association between pneumonia and prosthesis-wearing using fixed- and random-effects models, respectively. This finding suggests that there was substantial heterogeneity, and the true effect size might not be the same among included studies. Therefore, the random-effects meta-analysis was preferred in this review because of the high heterogeneity caused by the great variations of the study designs among the included studies, the intention to generalize the results beyond the included studies, and the number of the included studies was more than five [34].

Overall odds ratios of having pneumonia among prosthesis wearers were 1.43 (95% CI: 0.76 to 2.69) and 1.23 (95% CI: 1.07 to 1.42) using the random- and fixed-effects model, respectively. This result suggests that there was a potential increased trend of pneumonia occurring in the prosthesis-wearing group. However, the results should be interpreted cautiously, as a non-significant association was reported when using the random-effects model. Three included studies [9, 31, 32] reported a significantly increased risk of pneumonia for the prosthesis-wearing group, which is in contrast to the findings of Uno and Kubo [30]. The other two included studies [24, 29] showed no significant association between prosthesis-wearing and pneumonia. Today, the role of oral pathogens in the pathogenesis of pneumonia has been extensively reported [1–3]. Venkataraman et al. [35] found that the microbes residing intraorally are the primary driver of the lung microbiome. Therefore, the potential for respiratory bacteria, fungi, and viruses residing on prosthesis surfaces, oropharyngeal, and periodontal secretions could be the source of microorganisms aspirated into the respiratory system [2, 3, 36]. This susceptibility to microbial colonization is further increased by the prosthesis design requiring close and unpolished tissue-fitting surfaces that place the prostheses in direct and continuous contact with the oral mucosae and prosthesis hygiene practices [1, 37]. The diverse microbial community with the predominance of anaerobes on prosthesis surfaces may predispose prosthesis wearers to secondary coinfections or aggravate existing respiratory infections [14]. However, in the current review, the association of pneumonia and the prosthesis-wearing group did not achieve a conclusive finding when compared to non-prosthesis-wearing, which is consistent with van der Maarel-Wierink et al. [22]. Possibly, prosthesis cleanliness was not reported in most of the included studies in the present review. It is generally recognized that poor prosthesis hygiene [8], infrequent prosthesis cleaning [7], and nocturnal prosthesis wearing [6] were significantly associated with pneumonia. Therefore, this review provided evidence that prosthesis-wearing per se may not be significantly associated with pneumonia. Nonetheless, some studies have also reported a significantly reduced risk of aspiration pneumonia for the prosthesis-wearing group [20, 30]. Possibly, the swallowing mechanism improved after prosthesis-wearing due to the increased occlusal contact, subsequently reducing aspiration pneumonia. Notably, prosthesis hygiene and prosthesis-wearing habits for participants in the aforementioned studies were reported as satisfactory and primarily associated with aspiration pneumonia.

In studies assessing disease outcomes, the diagnostic criteria of pneumonia are important. All included studies in this review reported similar diagnostic criteria using validated medical records or diagnosed by physicians, but not self-reported. Therefore, subgroup analysis was performed to distinguish between the location of the origin of the etiologic infectious agents (community- or hospital-acquired). Estimated odds ratios for community-acquired pneumonia using random-effects was 1.43 (95% CI: 0.49 to 4.15). However, the removable prosthesis-wearing was significantly associated with community-acquired pneumonia, with an estimated odds ratio of 1.25 (95% CI: 1.08 to 1.45) when analysed using fixed-effects meta-analysis. Although the number of studies included in this analysis was fewer than 5, which suggests using a fixed-effects model [34], the heterogeneity was considerable. In addition, infrequent prosthesis cleaning among older adults increased the risk for community-acquired pneumonia (OR, 1.58; 95% CI, 1.15 to 2.17) [7]. However, the results of this study need to be interpreted with caution as it lacked a non-prothesis-wearing control group. A recent study by Alzamil et al. [9] found that prosthesis-wearing was a risk factor for community-acquired pneumonia. The results can be generalized to the bigger geriatric community, given the better study design, big sample size (2364 patients), and long follow-up time of up to 8 years. Possibly, a high prevalence of respiratory pathogens residing on unclean removable prostheses may be considered a major potential cause of respiratory infections in community-dwelling older adults [15, 38].

In this systematic review, the certainty of the evidence evaluated using the GRADE approach was very low. There were five included cohort studies that presented with inconsistency and imprecision, which was related to high heterogeneity, minimal or no overlap of confidence intervals, and wide confidence intervals. This might also be related to the variation of the study designs and the nature of pneumonia which may be associated with various risk factors [3]. However, it is not a simple task to exclude all confounding factors. Therefore, subgroup analyses were performed in the present review in order to reduce distortion.

The limitation of this review was the high statistical heterogeneity of the meta-analyses due to various outcome variables, resulting in a difficult mutual comparison of the results. Therefore, future studies with bigger sample sizes, longer follow-ups, reduced confounding factors, and more prospective, controlled study designs investigating the relationship between removable prosthesis-wearing or hygiene and pneumonia are strongly recommended to overcome the limitations of the existing evidence. Additionally, the application of language restriction was also considered a limitation in this systematic review. Studies published in other languages should be considered in future reviews to reduce the risk of a biased summary effect.

## Conclusions

Based on the outcomes of the non-randomized clinical studies in this systematic review, there was no definitive evidence to substantiate the notion that prosthesis-wearing contributed to the risk of developing pneumonia. All included studies, assessed using the Newcastle-Ottawa Scale, were considered to be of good quality. However, the certainty of the evidence was low to very low. Therefore, future clinical studies are recommended to investigate the association between prosthesis-wearing and pneumonia in order to overcome the limitations of the existing evidence.

## Electronic supplementary material

Below is the link to the electronic supplementary material.

Supplementary Material 1

## Data Availability

All data generated or analysed during this study are included in this published article and additional files.

## Abbreviations

CI: Confidence interval
EMBASE: Excerpta medica dataBASE
GRADE: Grading of recommendations, assessment, development and evaluation
MEDLINE: Medical literature analysis and retrieval system online
NOS: Newcastle-Ottawa scale
OR: Odds ratio
PECOS: population, intervention/ exposure, outcome, and study
PROSPERO: International prospective register of systematic reviews
